# Primary Health Care Workforce in Southeast Asia Region, Existing Status and Strategies for Non-Communicable Diseases and Oral Health Alliance: A Scoping Review

**DOI:** 10.7759/cureus.22362

**Published:** 2022-02-18

**Authors:** Surbhi Kapoor, Vikrant Mohanty, Aswini Y. Balappanavar, Puneet Chahar, Kavita Rijhwani

**Affiliations:** 1 Public Health Dentistry, Maulana Azad Institute of Dental Sciences, New Delhi, IND

**Keywords:** oral health education, primary health care, chronic diseases, oral health, community health workers

## Abstract

Oral diseases and non-communicable diseases (NCD) share modifiable risk factors and common social determinants, thus creating new opportunities for improving oral health. The existing primary health care workers can play an integral role in NCD and oral health care integration by creating awareness, promoting oral health, controlling risk factors, and referring for timely dental care. This study aimed to identify and understand the roles of the existed human health resources working at primary health care and develop strategies to build on a unified NCD and oral health alliance human resources at this level.

A scoping review was conducted to identify the primary health workforce in the Southeast Asia region, their roles and responsibility, and integration in oral health care. Various databases like NCBI (PubMed), Google Scholar, World Health Organisation (WHO)-Southeast Asia region (SEAR), Ministry of Health and Family Welfare websites in SEAR were comprehensively searched from January 1980 to December 2020 for reports, reviews, and original research. The keywords used were “primary health care workers,” “community health care workers,” “primary oral health care in SEAR,” etc. Only full-text articles in English language and reports available in Ministry of Health and Family Welfare (MOHFW) sites of SEAR were included in the study.

Information was taken from 39 full-text articles, six WHO reports, and two reports from Ministry of Health sites of SEAR. Primary health workers (PHW) are known by multiple names in different countries of Southeast Asia. They share various common roles and responsibilities. There are many opportunities for the prevention and control of oral diseases in the SEAR. Basic systems and human resources for the control of NCD are in place in many countries. Oral health can be integrated with NCD programs and policies to reduce the burden of diseases.

## Introduction and background

Non-communicable diseases (NCDs) are widely identified as a key challenge to health and sustainable human development. NCDs are the leading cause of death and disability worldwide, responsible for 70% of global mortality [1]. The low- and middle-income countries (LMICs) are most likely to be affected by these highly preventable diseases. World Health Organisation (WHO) has recognized the following four major non-communicable diseases: cardiovascular disease (CVD), cancer, diabetes, and chronic respiratory diseases; and four major risk factors as follows: tobacco use, unhealthy diet, physical inactivity, and harmful use of alcohol [2]. There exists a range of diseases that share common risk factors and are also linked to the four most common NCDs. Oral diseases have similar risk factors and are highly interconnected [3]. There is an urgent need to reorganize our health systems so that integrated approaches can be implemented rather than a single disease approach.

Oral diseases and NCDs share modifiable risk factors and common social determinants, thus creating new opportunities for improving oral health [3]. The various risk factors that have an impact on oral health are as follows: diet high in sugars can lead to tooth decay, tobacco use and poor diet are etiological agents of periodontal diseases, and tobacco and alcohol use are strongly implicated in the development of oral and oropharyngeal cancer [4]. Oral disease affects the overall quality of life of an individual like pain and discomfort, difficulty in chewing, loss of school hours, low self-esteem, and economic loss. Across the globe, the prevalence of people suffering from oral diseases is 3467 million [5]. The oral disease burden is high in the Southeast Asia region (SEAR) [6].

Improving oral health will have a significant impact on reducing morbidity and mortality from NCD. WHO has developed the basic package of oral care (BPOC) as a complement to its package of essential non-communicable disease interventions (WHO-PEN) for primary health care in low-resource settings [7]. Effective strategies if implemented in SEAR to promote oral health and prevent oral diseases can contribute to preventing the leading NCDs. Stronger health systems supported by primary health care (PHC) are crucial to effectively managing NCDs and their risk factors. Hence, the integration of oral health with NCD at the primary level will be a crucial strategy.

“Primary health care” is the first level of contact where essential health care services are provided to an individual. Primary health care (PHC) is an approach to achieve both the millennial developmental goals and the wider goal of universal health coverage to health through acceptable, accessible, appropriate, and affordable health care [8]. The wide variety of preventive and curative services provided within PHC acts as a cost-effective strategy, especially for middle- and low-income countries. PHC has been incorporated into the national health policy and strategy in all countries of the Southeast Asia (SEA) regions [9]. The existing primary health care workers can play an integral role in NCD and oral health care integration by creating awareness, promoting oral health, controlling risk factors, and referring for timely dental care. There is no comprehensive review of how primary health care workforce is engaged in the integration of oral health into wider NCD prevention and control. With this background, the primary objective of this study was to identify and understand the various roles of the existed human health resources working at primary health care play in NCD prevention and control. The secondary objective was to develop strategies to build on a unified NCD and oral health alliance human resources at the primary health care level.

## Review

Methodology

A scoping review was conducted to identify the primary health workforce in the Southeast Asia region, their roles and responsibility. Various strategies have been identified and formulated to integrate oral health in general health.

Search Strategy

Various databases like NCBI (PubMed), Google Scholar, Ministry of Health (MOH) sites of all SEAR, and WHO sites were comprehensively searched for reports, reviews, and original research from January 1980 till December 2020. The corresponding author (SK) collected the data. Designing of the study and preparation of the manuscript were done by the corresponding author (SK) along with two other authors (VK and PC). The other two authors (AYB and KR) helped in analysis and results formulation. Full-text articles in English language and reports available in Ministry of Health sites of SEAR were the research works included in this study. The list of keywords and combination of keywords as well as medical subject heading (MeSH) terms are provided in Table 1 below.

**Table 1:** Search strategy from various databases SEAR: Southeast Asia region; MeSH: medical subject heading

Keywords	MeSH terms
“Primary oral health care,” “primary health care workers, “primary health care workers in SEAR,” “community health care workers in SEAR,” “ primary health care workers and SEAR,” “community health care workers and sear,” “oral health integration in primary health care”	“Primary health care,” “community health workers,” “community health workers/statistics and numerical data,” “community health workers/trends”

Results

The search yielded 1276 articles identified from electronic databases and 115 articles were identified through a reference list. After removing the duplicates, the articles were screened based on eligibility criteria. Information was taken from 39 full-text articles, six WHO reports, and two reports from Ministry of Health sites of SEAR.

Primary Health Workers in SEARO

The Southeast Asia region consists of 11 countries: India, Bhutan, Myanmar, Bangladesh, North Korea, Indonesia, Maldives, Nepal, Sri Lanka, Thailand, and Timor Leste. The number of health workers for PHC varies from 500 in Timor-Leste to 264,368 persons in India. The ratio of health workers for PHC services to population varies from 0.3 (India and Myanmar) to 2.5 (Maldives) per 1000 populations. The range of available numbers of categories for PHC workers varies from 18 categories in Bangladesh to three-four categories in Bhutan, North Korea, and Thailand (Table 2) [10].

**Table 2:** Number of community health workers in SEAR countries SEAR: Southeast Asia region Source: Global health observatory data repository WHO [11].

SEAR country	Numbers
Bangladesh (2018)	55,136
Bhutan (2012)	578
Maldives (2014)	509
India (2018)	970,676
Myanmar (2015)	2326
Nepal (2004)	16,206
Thailand (2016)	13,006
Timor-Leste (2013)	241

Primary Health Care Workers and Their Responsibilities

Primary health workers (PHW) are known by multiple names in different countries of Southeast Asia. They are referred to as village health volunteers in Thailand [12], mobile barefoot doctors in Nepal [13], basic health workers in Bhutan [14], accredited social health activists (ASHAs) and health workers and health assistants in India [15], kaders in Indonesia, etc [16]. They share various common roles and responsibilities like ensuring safe water supply, food supply to malnourished children, drug depots, health education and promotion, disease control, communicable disease surveillance, immunization, first aid to minor ailments, etc. In the majority of Southeast Asia regions (Myanmar, Bhutan, Korea, India, Indonesia, and Nepal), maternal and child health care and family planning activities in primary health care centers are taken care of by auxiliary nurse midwives [10,13-15]. In addition to auxiliary nurse midwives, other primary health workers are involved in mother and child care services. The human health resources and their roles and responsibilities are given in Table 3.

**Table 3:** Primary human resources in SEAR and their roles and responsibilities SEAR: Southeast Asia region; NA: information not available; ASHA: accredited social health activist; ANM: auxiliary nurse midwife; T.B.: tuberculosis; DP: democratic party

Country	Economic status of country [17]	General responsibilities [10,12-15,18]	Maternal child care [10,13,15,19]	Health education [10]	Drug [10,20,21]	T.B. [22-24]	Mental health [25-27]
Bangladesh	Low-middle	NA	Health inspector, health assistant, Upazila family planning officer, family welfare visitor, family welfare assistant	Medical officer, sanitary inspector, health inspector, assistant health inspector, family welfare visitor, nursing supervisor, senior staff nurse	Assistant health inspectors	Community health workers	NA
Bhutan	Low- middle	Basic Health Worker, Health Assistants	Auxiliary nurse midwives, health assistant and basic health worker	Auxiliary nurse and midwife, basic health worker	NA	NA	NA
Nepal	Low	Mobile barefoot doctors, community health leader, assistant health worker and senior nurse	Auxiliary nurse midwives, maternal child health worker, village health worker, assistant health worker, senior nurse	Village health worker, assistant health worker, senior nurse, ANM	NA	Village health worker	NA
Maldives	Upper- middle	NA	NA	Senior medical officer, medical officer, staff nurse, community health worker, nurse aid, laboratory technician, lab assistant trainee, family health worker, traditional birth attendant	Community health workers, family health workers, foolhumaas	NA	NA
Thailand	Upper-middle	Village health volunteers along with technical nurse, registered nurse, public health officer	NA	Technical nurse, registered nurse, public health officer, village health volunteer	NA	NA	Village health volunteer
Indonesia	Low-middle	Kaders	Auxiliary nurse midwives, medical officer	Medical officer, health promotion officer	NA	TB nurses	Community health workers
India	Low-middle	ASHA	Auxiliary nurse midwives, ASHA, Anganwadi worker, medical officer, health assistant female and male	Medical officer, block extension educator, lady health visitor, health assistant male, ANM, ASHA	ASHA	ASHA	
Sri Lanka	Low-middle	NA	Public health midwives and in Maldives traditional birth attendant	Divisional director of health services, supervising public health inspector, public health inspector, public health nursing sister, supervising public health midwives, public health midwives	NA	NA	Community mental health workers
Myanmar	Low-middle	Community health worker	Auxiliary nurse midwives, township health assistant, township health nurse, health assistant, health assistant, public health supervisor, public health supervisor, lady health visitor	Township health assistant, township health nurse, health assistant-1, health assistant-2, public health supervisor-1, public health supervisor-2, lady health visitor midwives	NA	NA	NA
Timor-Leste	Low-middle	NA	NA	NA	NA	Community health care workers	NA
North Korea	Low	NA	Auxiliary nurse midwives, nurse	Household doctor, nurse, midwives	Nurse	NA	NA

Strategies of Oral Health Integration in Other Countries

General health care and oral health care systems have generally evolved separately all over the world in the last 150 years [4,27-29]. Oral health care is often only partially integrated into public health care systems, or it is entirely absent. Integrated approaches are the most cost-effective and realistic way to eliminate this problem [30]. An ideal primary (oral) health care system should provide universal coverage, be people-centered, have demand-led policies and programs, and be integrated with general health in all policies, including labor, environment, and education [31].

High-Income Countries

Oral health care integration into primary care has been implemented by many developed and few developing countries. In countries like USA and Australia, strategies for oral health integration into primary care are oriented towards oral health clinical competencies achievement for non-dental primary care providers [32]. PHW integration in oral health education and promotion represents a promising approach. PHW training curriculum targeting pediatric oral health promotion has been established by many countries. Action for dental health was initiated by the American Dental Association to deploy a new type of PHW that has a focus on patient education, disease prevention, and patient navigation. A Healthy Mouth for a Lifetime: Beyond the Basic by Dental Health, an initiative of San Diego trains community members to be a dental health resource for individuals, including parents and older adults [33]. Canada has initiated a community-based dental health program for children and caregivers in remote communities in Canada. The PHW collaborate with externally contracted dental therapists and dental hygienists to screen zero-to-seven-year-old children for dental caries, provide preventive oral health information, apply fluoride varnish and refer children to the dental therapist or hygienist for sealants and atraumatic restorative treatment (ART) or to a dentist for further complex treatment, as needed [34]. 

Low- and Middle-Income Countries

The policymakers in Brazil have implemented population-based models such as “smiling Brazil,” the Brazilian National Oral Health Policy (PNSB) [35]. In the small towns of Piauí State of Brazil, communitarian agents of health have undertaken many activities related to oral health. They perceive that they have a fair knowledge of oral health and perform activities related to dental care [36]. Lay health counsellor in Vietnam provided in-person counseling about healthy oral cavities and incentives to mothers of newborns. They discuss the importance of tooth cleaning, teething, and baby bottle tooth decay [37].

Scenario of SEAR

Oral health services are limited and are unavailable to many, either due to low availability and accessibility of oral health care or because oral health care is costly. There is a lack of integration of oral health care and oral health workforce in primary health care in the majority of SEAR countries.

A separate National Oral Health Policy was available for Nepal, and it is in its developmental stage in India. The National Oral Health Policy of Nepal has developed 14 key strategies for the upliftment of oral health care in their country. One of the key strategies is to expand oral health care to rural communities by developing training programs for existing CHW [38]. In India, the government has taken several steps for the integration of oral health with medical health. National Oral Health Program under MOH, Government of India, aims of providing optimal oral health care. This program had several objectives, one among them was the integration of oral health promotion and preventive services with the general health care system [39]. Few countries have a dental officer as oral health incharge of some of primary health care centers like Thana Health Complex in Bangladesh [19], Puskesmas in Indonesia [40], and PHC in Sri Lanka [41]. Dental officers in some states of India are appointed in PHC centers through Combined Medical Services Examination conducted by UPSC [42]. 

Integration Framework in SEAR

Multisectoral collaborations are vital for the reduction of oral disease burden. The global action plan of WHO for the prevention and control of non-communicable diseases can integrate oral health preventive control approaches [43].

Reducing the risk factors of oral health and other NCD through health promotion and primary prevention: NCD and oral diseases share common risk factors like tobacco use, unhealthy diet, physical inactivity, and harmful use of alcohol. They can largely be prevented by addressing the four common risk factors. Several population-based strategies such as information, education, and communication (IEC) material development, awareness programs, lectures, radio broadcast,s and television advertisements can be implemented. The primary health worker is an integral part of his community and is seen as a role model or as a healer. Therefore, primary health care workers can be used to disseminate this information to the grassroots level. Involvement of all members of the community and bringing a sustained behavior change should be the key strategy. Health promotion is not enough legislation will also play a vital role. One such example is the WHO Framework Convention on Tobacco Control (FCTC) is the first legally binding international treaty to reduce harm due to tobacco. In the SEA region, all countries except Indonesia have ratified the FCTC and are implementing the various elements of the MPOWER package [44]. Similar policies are needed like increased taxation to reduce the demand for other unhealthy products such as sugary drinks; conversely, subsidies should be provided on fruits and vegetables.

Early detection and management of oral diseases and other NCD: All countries have national programs for early detection of NCDs, e.g., in India, the National Program for Prevention and Control of Cancer, Diabetes, Cardiovascular Diseases, and Stroke. In the SEA region, pilot projects for integrating NCDs within the primary health care system are underway in Bhutan and Sri Lanka and are planned in a few other countries. Primary health care workers can also be trained to screen for initial signs and symptoms of dental diseases, such as white spot lesion or black discoloration of teeth for caries, bleeding of gums or oral mal-odor for gingival and periodontal diseases, red and white lesions for premalignant lesions, and screen for tobacco stains on teeth. Training material can be designed for CHW each country in their regional language just like dental resource material developed by ADA and San Diego. Just like drug kits available to ASHA in India, an oral kit can also be provided. The oral kit may include oral cavity models, mouth mirrors, cotton, etc. for the detection of dental diseases. Referral systems can be organized to those identified with lesions or any dental diseases.

Delivering oral health and other NCD interventions using the primary health care approach: The primary health workers can be trained for a period of three to six months by dentists to provide preventive services to their community. Fluoride mouth rinsing activities should be implemented by primary health care workers among school children. Since fluoride promotes the remineralization of enamel services. Live Learn Laugh program was launched through a public-private partnership between Fédération Dentaire Internationale and Unilever with a goal to improve oral health by encouraging daily tooth brushing. Primary health care workers can execute such programs in SEAR [45]. Fluoride gel application can also be done in patients with high risk of developing dental decay. ART services that are part of the Basic Package of Oral Care by FDI should be included as primary care services in PHC [46]. Hence, this treatment modality should be implemented in order to reduce the burden of dental caries.

Surveillance and research to quantify and track oral health and NCD integration their determinants: The surveillance policies should be implemented to keep a track of the common risk factors, morbidity, and mortality from these diseases. Government should encourage research work in areas of NCD and oral health by health care institutes. NCD and oral health integration involving primary health workers is a crucial strategy to reduce the burden of the diseases. Various factors are essential for successful integration like political priorities, stringent policies, capacity building for primary health care workers, ensuring availability of essential and affordable medicines and technologies, and financial support for health sector.

Limitations

The study generated evidence on the current status of primary health workforce in Southeast Asian countries; however, it had its limitations as the data about any existing roles and responsibilities of the primary health care workforce was not adequate which may not reflect their actual status with respect to oral health care service delivery. Further, the current study was a scoping review, therefore quality check of articles included was not part of this study.

## Conclusions

Oral disease burden is enormously high in SEAR and other countries as well. Oral diseases and NCD share many common risk factors. Hence, there are many opportunities for the prevention and control of oral diseases in the SEAR. Basic systems and human resources for the control of NCD are in place in many countries. Oral health can be integrated with NCD programs and policies to reduce the burden of all diseases.

Universal oral health coverage remains an aspiration for all. Trained PHC workers can be effectively utilized for the integration of oral health into general health. A dedicated workforce along with adequate funding, integrated health system planning, and support from policymakers can reform the goal of affordable and accessible oral health care.
